# *catena*-Poly[(acetato­chloridozinc)-μ-1,1′-[1,4-phenyl­enebis(methyl­ene)]di-1*H*-imidazole]

**DOI:** 10.1107/S1600536813000524

**Published:** 2013-01-12

**Authors:** Liang Wang

**Affiliations:** a Department of Chemistry, University of Science and Technology Beijing, Beijing 100083, People’s Republic of China

## Abstract

The title compound, [Zn(CH_3_CO_2_)Cl(C_14_H_14_N_4_)]_*n*_, is a one-dimensional coordination polymer in which the Zn^II^ ion is tetrahedrally ­coordinated by two N atoms of a bridging 1,1′-[1,4-phenyl­enebis(methyl­ene)]di-1*H*-imidazole ligand, an acetate O atom and a Cl atom. The Cl atom, two acetate O atoms and two acetate C atoms are located on a mirror plane. The coordination of the diimidazole ligand to the Zn^II^ ion gives an infinite one-dimensional zigzag structure along the *b*-axis direction with the charge balanced by the chloride and acetate ions.

## Related literature

For background to the design and assembly of metal-organic coordination polymers, see: Wang *et al.* (2009 ▶); Leininger *et al.* (2000 ▶). For a related structure, see: Li *et al.* (2008 ▶). For the synthesis of the title complex, see: Wang *et al.* (2012 ▶). 
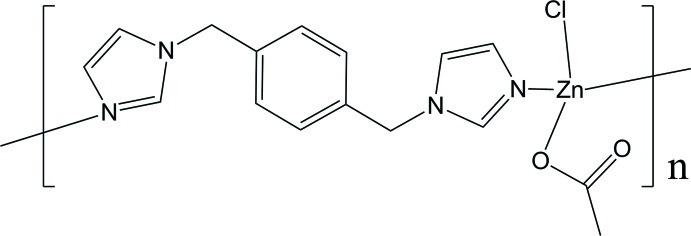


## Experimental

### 

#### Crystal data

[Zn(C_2_H_3_O_2_)Cl(C_14_H_14_N_4_)]
*M* *_r_* = 398.18
Monoclinic, 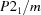

*a* = 7.4510 (5) Å
*b* = 14.1636 (8) Å
*c* = 8.1977 (5) Å
β = 90.459 (6)°
*V* = 865.10 (9) Å^3^
*Z* = 2
Mo *K*α radiation
μ = 1.59 mm^−1^
*T* = 293 K
0.20 × 0.15 × 0.10 mm

#### Data collection

Bruker APEXII CCD area-detector diffractometer
Absorption correction: multi-scan (*SADABS*; Sheldrick, 2003 ▶) *T*_min_ = 0.742, *T*_max_ = 0.857
5952 measured reflections
1867 independent reflections
1375 reflections with *I* > 2σ(*I*)
*R*_int_ = 0.076

#### Refinement

*R*[*F* ^2^ > 2σ(*F* ^2^)] = 0.054
*wR*(*F* ^2^) = 0.124
*S* = 1.00
1867 reflections
118 parameters
H-atom parameters constrained
Δρ_max_ = 0.55 e Å^−3^
Δρ_min_ = −0.50 e Å^−3^

### 

Data collection: *APEX2* (Bruker, 2004 ▶); cell refinement: *SAINT-Plus* (Bruker, 2001 ▶); data reduction: *SAINT-Plus*; program(s) used to solve structure: *SHELXS97* (Sheldrick, 2008 ▶); program(s) used to refine structure: *SHELXL97* (Sheldrick, 2008 ▶); molecular graphics: *XP* in *SHELXTL* (Sheldrick, 2008 ▶); software used to prepare material for publication: *SHELXTL*.

## Supplementary Material

Crystal structure: contains datablock(s) global, I. DOI: 10.1107/S1600536813000524/hp2053sup1.cif

Structure factors: contains datablock(s) I. DOI: 10.1107/S1600536813000524/hp2053Isup2.hkl

Additional supplementary materials:  crystallographic information; 3D view; checkCIF report

## Figures and Tables

**Table 1:** Selected bond lengths (Å)

Zn1—O1	1.957 (4)
Zn1—N1	2.014 (3)
Zn1—Cl1	2.2428 (17)

**Fig. 1.**
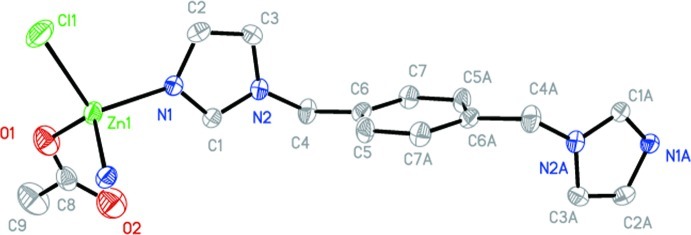
Molecular structure with the unique atom-labelling scheme. Displacement ellipsoids are drawn at the 30% probability level, hydrogen atoms are omited for clarity.

**Fig. 2.**
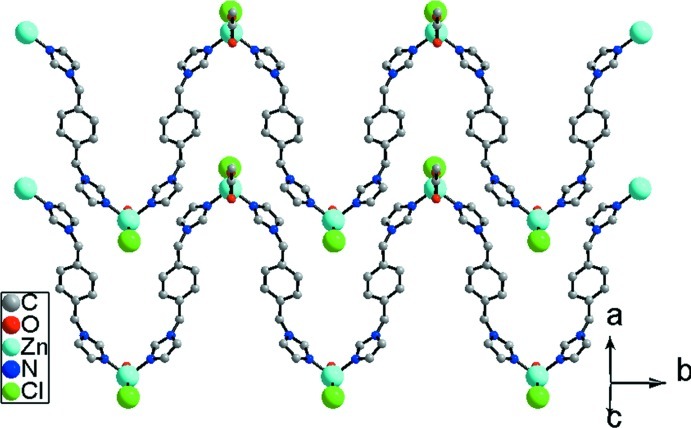
A view of two-dimensional supramolecular diagram.

## supplementary crystallographic information

### Comment

In the field of supramolecular chemistry and crystal engineering, the design and assembly of metal-organic coordination polymers with appealing structures and properties have stimulated interests of chemists in recent years (Wang *et al.*, 2009 and Leininger *et al.* 2000)). Thus far, a large number of metal-organic coordination polymers have been prepared. Herein, a new metal coordination polymer has been prepared by using a bidentate ligand.

As shown in Fig. 1, the asymmetric unit of compound was composed of a 1,4-bis((1*H*-imidazol-1-yl)methyl)benzene ligand (*L*), a divalent zinc ion, a chloride ion, and an acetate ion. The bond lengths of Zn—N, Zn—O, and Zn—Cl are consistent with those reported result (Li *et al.*, 2008). The orgainic ligand in a *trans*-coordination mode to coordinate with the zinc ions by using two terminal nitrogen atoms, leading to the one-dimensional zigzag coordination polymer. The dihedral angle of the imidazol and benzene planes is 87.293 (2)°, and two the imidazol planes is parallel with the dihedral angle of 0°. The adjacent single chains are parallel along the direction of *b* axis, Fig. 2.

### Experimental

The title compound was synthesized referring to the reported literature (Wang *et al.*, 2012). A mixture of Zn(OAc)_2_.2H_2_O (0.0422 g, 0.1 mmol), ZnCl_2_ (0.014 g, 0.1 mmol), and 1,4-bis((1*H*-imidazol-1-yl)methyl)benzene ligand (*L*) (0.024 g, 0.1 mmol), and H_2_O (15 ml) was sealed in 25 ml Teflon-lined stainless steel reactor and heated to 120 *^o^*C. Colorless block-shaped crystals suitable for X-ray diffraction analysis were separated by filtration with the yield of 0.031 g, 78% (based on ligand).

### Refinement

All the non-hydrogen atoms were refined anisotropically by full-matrix leastsquares calculations on F^2^. All H atoms (except H1a) were placed in geometrically idealized positions and treated as riding on their parent atoms with C—H = 0.93 Å, *U*_iso_ = 1.2Ueq (C) for aromatic atoms, C—H = 0.96 Å, C—H = 0.96 Å, *U*_iso_ = 1.5Ueq (C) for methyl atoms.

### Crystal data

**Table:**

[Zn(C_2_H_3_O_2_)Cl(C_14_H_14_N_4_)]	*F*(000) = 408
*M**_r_* = 398.18	*D*_x_ = 1.529 Mg m^−^^3^
Monoclinic, *P*2_1_/*m*	Mo *K*α radiation, λ = 0.71073 Å
Hall symbol: -P 2yb	Cell parameters from 1643 reflections
*a* = 7.4510 (5) Å	θ = 2.9–29.0°
*b* = 14.1636 (8) Å	µ = 1.59 mm^−^^1^
*c* = 8.1977 (5) Å	*T* = 293 K
β = 90.459 (6)°	Block, colorless
*V* = 865.10 (9) Å^3^	0.20 × 0.15 × 0.10 mm
*Z* = 2	

### Data collection

**Table:**

Bruker APEXII CCD area-detector diffractometer	1867 independent reflections
Radiation source: fine-focus sealed tube	1375 reflections with *I* > 2σ(*I*)
Graphite monochromator	*R*_int_ = 0.076
φ and ω scans	θ_max_ = 26.5°, θ_min_ = 2.9°
Absorption correction: multi-scan (*SADABS*; Sheldrick, 2003)	*h* = −8→9
*T*_min_ = 0.742, *T*_max_ = 0.857	*k* = −17→17
5952 measured reflections	*l* = −10→6

### Refinement

**Table:**

Refinement on *F*^2^	Primary atom site location: structure-invariant direct methods
Least-squares matrix: full	Secondary atom site location: difference Fourier map
*R*[*F*^2^ > 2σ(*F*^2^)] = 0.054	Hydrogen site location: inferred from neighbouring sites
*wR*(*F*^2^) = 0.124	H-atom parameters constrained
*S* = 1.00	*w* = 1/[σ^2^(*F*_o_^2^) + (0.0537*P*)^2^] where *P* = (*F*_o_^2^ + 2*F*_c_^2^)/3
1867 reflections	(Δ/σ)_max_ = 0.001
118 parameters	Δρ_max_ = 0.55 e Å^−^^3^
0 restraints	Δρ_min_ = −0.50 e Å^−^^3^

### Special details

**Table:**

Geometry. All e.s.d.'s (except the e.s.d. in the dihedral angle between two l.s. planes) are estimated using the full covariance matrix. The cell e.s.d.'s are taken into account individually in the estimation of e.s.d.'s in distances, angles and torsion angles; correlations between e.s.d.'s in cell parameters are only used when they are defined by crystal symmetry. An approximate (isotropic) treatment of cell e.s.d.'s is used for estimating e.s.d.'s involving l.s. planes.
Refinement. Refinement of *F*^2^ against ALL reflections. The weighted *R*-factor *wR* and goodness of fit *S* are based on *F*^2^, conventional *R*-factors *R* are based on *F*, with *F* set to zero for negative *F*^2^. The threshold expression of *F*^2^ > σ(*F*^2^) is used only for calculating *R*-factors(gt) *etc*. and is not relevant to the choice of reflections for refinement. *R*-factors based on *F*^2^ are statistically about twice as large as those based on *F*, and *R*- factors based on ALL data will be even larger.

### Fractional atomic coordinates and isotropic or equivalent isotropic displacement parameters (Å^2^)

**Table:**

	*x*	*y*	*z*	*U*_iso_*/*U*_eq_	Occ. (<1)
C1	1.1006 (5)	0.1143 (2)	0.9055 (4)	0.0387 (9)	
H1	1.1791	0.1472	0.9735	0.046*	
C2	0.8642 (5)	0.0711 (2)	0.7765 (5)	0.0441 (9)	
H2	0.7465	0.0693	0.7379	0.053*	
C3	0.9946 (6)	0.0095 (2)	0.7356 (5)	0.0443 (10)	
H3	0.9840	−0.0419	0.6657	0.053*	
C4	1.3238 (5)	−0.0061 (3)	0.8149 (5)	0.0483 (10)	
H4A	1.3126	−0.0715	0.8485	0.058*	
H4B	1.4013	0.0255	0.8934	0.058*	
C5	1.4064 (6)	0.0774 (2)	0.5539 (5)	0.0448 (10)	
H5	1.3423	0.1299	0.5888	0.054*	
C6	1.4113 (5)	−0.0027 (2)	0.6486 (4)	0.0372 (9)	
C7	1.5034 (5)	−0.0802 (3)	0.5934 (5)	0.0438 (10)	
H7	1.5053	−0.1351	0.6554	0.053*	
C8	0.9449 (11)	0.2500	1.2806 (8)	0.0513 (16)	
C9	0.9467 (13)	0.2500	1.4712 (9)	0.094 (3)	
H9A	1.0686	0.2500	1.5101	0.140*	
H9B	0.8864	0.1947	1.5103	0.140*	0.50
H9C	0.8864	0.3053	1.5103	0.140*	0.50
O1	0.8030 (8)	0.2500	1.2164 (5)	0.0686 (13)	
O2	1.0927 (8)	0.2500	1.2147 (6)	0.0856 (16)	
Zn1	0.80781 (9)	0.2500	0.97775 (7)	0.0402 (2)	
N1	0.9317 (4)	0.13638 (19)	0.8831 (4)	0.0374 (7)	
N2	1.1458 (4)	0.03823 (19)	0.8181 (3)	0.0373 (7)	
Cl1	0.5193 (2)	0.2500	0.8976 (3)	0.0729 (5)	

### Atomic displacement parameters (Å^2^)

**Table:**

	*U*^11^	*U*^22^	*U*^33^	*U*^12^	*U*^13^	*U*^23^
C1	0.032 (2)	0.045 (2)	0.039 (2)	−0.0021 (17)	0.0033 (17)	−0.0050 (16)
C2	0.031 (2)	0.049 (2)	0.052 (2)	−0.0049 (18)	−0.0030 (18)	0.0016 (19)
C3	0.044 (3)	0.041 (2)	0.048 (2)	−0.0033 (19)	0.0000 (19)	−0.0112 (18)
C4	0.041 (2)	0.061 (2)	0.043 (2)	0.015 (2)	0.0049 (19)	0.0020 (19)
C5	0.041 (2)	0.0401 (19)	0.053 (2)	0.0126 (18)	0.0107 (19)	−0.0021 (18)
C6	0.028 (2)	0.0403 (19)	0.044 (2)	0.0007 (16)	0.0017 (16)	0.0010 (17)
C7	0.046 (3)	0.0381 (19)	0.047 (2)	0.0068 (18)	0.0048 (19)	0.0060 (17)
C8	0.066 (5)	0.031 (3)	0.057 (4)	0.000	0.016 (4)	0.000
C9	0.136 (9)	0.091 (5)	0.054 (4)	0.000	0.000 (5)	0.000
O1	0.090 (4)	0.067 (3)	0.049 (3)	0.000	0.014 (3)	0.000
O2	0.094 (5)	0.084 (3)	0.079 (4)	0.000	0.001 (3)	0.000
Zn1	0.0355 (4)	0.0390 (4)	0.0464 (4)	0.000	0.0116 (3)	0.000
N1	0.0322 (18)	0.0369 (15)	0.0431 (18)	−0.0003 (14)	0.0047 (14)	−0.0040 (14)
N2	0.0350 (19)	0.0415 (16)	0.0355 (16)	0.0040 (14)	0.0056 (14)	0.0007 (14)
Cl1	0.0332 (9)	0.0714 (10)	0.1140 (16)	0.000	0.0032 (9)	0.000

### Geometric parameters (Å, º)

**Table:**

C1—N1	1.308 (5)	C5—H5	0.9300
C1—N2	1.338 (4)	C6—C7	1.372 (5)
C1—H1	0.9300	C7—C5^i^	1.387 (5)
C2—C3	1.350 (5)	C7—H7	0.9300
C2—N1	1.366 (4)	C8—O1	1.177 (8)
C2—H2	0.9300	C8—O2	1.231 (8)
C3—N2	1.371 (5)	C8—C9	1.562 (9)
C3—H3	0.9300	C9—H9A	0.9600
C4—N2	1.468 (5)	C9—H9B	0.9600
C4—C6	1.516 (5)	C9—H9C	0.9600
C4—H4A	0.9700	Zn1—O1	1.957 (4)
C4—H4B	0.9700	Zn1—N1	2.014 (3)
C5—C6	1.375 (5)	Zn1—N1^ii^	2.014 (3)
C5—C7^i^	1.387 (5)	Zn1—Cl1	2.2428 (17)
			
N1—C1—N2	111.3 (3)	C5^i^—C7—H7	119.6
N1—C1—H1	124.3	O1—C8—O2	127.4 (7)
N2—C1—H1	124.3	O1—C8—C9	116.6 (7)
C3—C2—N1	109.5 (3)	O2—C8—C9	116.0 (7)
C3—C2—H2	125.2	C8—C9—H9A	109.5
N1—C2—H2	125.2	C8—C9—H9B	109.5
C2—C3—N2	106.1 (3)	H9A—C9—H9B	109.5
C2—C3—H3	127.0	C8—C9—H9C	109.5
N2—C3—H3	127.0	H9A—C9—H9C	109.5
N2—C4—C6	113.4 (3)	H9B—C9—H9C	109.5
N2—C4—H4A	108.9	C8—O1—Zn1	115.1 (5)
C6—C4—H4A	108.9	O1—Zn1—N1	113.41 (12)
N2—C4—H4B	108.9	O1—Zn1—N1^ii^	113.41 (12)
C6—C4—H4B	108.9	N1—Zn1—N1^ii^	106.09 (17)
H4A—C4—H4B	107.7	O1—Zn1—Cl1	105.52 (18)
C6—C5—C7^i^	120.3 (3)	N1—Zn1—Cl1	109.17 (9)
C6—C5—H5	119.9	N1^ii^—Zn1—Cl1	109.17 (9)
C7^i^—C5—H5	119.9	C1—N1—C2	106.1 (3)
C7—C6—C5	118.9 (3)	C1—N1—Zn1	125.5 (2)
C7—C6—C4	119.4 (3)	C2—N1—Zn1	128.3 (2)
C5—C6—C4	121.6 (3)	C1—N2—C3	107.0 (3)
C6—C7—C5^i^	120.8 (3)	C1—N2—C4	125.8 (3)
C6—C7—H7	119.6	C3—N2—C4	127.1 (3)

Symmetry codes: (i) −*x*+3, −*y*, −*z*+1; (ii) *x*, −*y*+1/2, *z*.
